# Pneumocystis pneumonia in South African children diagnosed by molecular methods

**DOI:** 10.1186/1756-0500-7-26

**Published:** 2014-01-10

**Authors:** Brenda M Morrow, Catherine M Samuel, Marco Zampoli, Andrew Whitelaw, Heather J Zar

**Affiliations:** 1 Department of Paediatics and Child Health, Red Cross War Memorial Children’s Hospital (RCWMCH), University of Cape Town, 5th Floor Institute of Child Health Building, Klipfontein Road, Rondebosch 7700, Cape Town, South Africa; 2 Division of Medical Microbiology, National Health Laboratory Services, University of Cape Town, Cape Town, South Africa

**Keywords:** Pneumocystis pneumonia, HIV, Children, Prophylaxis, PCR, Diagnosis, Incidence

## Abstract

**Background:**

*Pneumocystis* pneumonia (PCP) is an important cause of hospitalization and mortality in HIV-infected children. However, the incidence of PCP has been underestimated due to poor sensitivity of diagnostic tests. The use of polymerase chain reaction (PCR) for pneumocystis has enabled more reliable diagnosis. This study describes the incidence, clinical features and outcome of PCP in South African children diagnosed using PCR.

**Methods:**

A prospective study of children hospitalised in South Africa with suspected PCP was done from November 2006 to August 2008. Clinical, laboratory and radiological information were collected. Lower respiratory tract specimens were obtained for PCP immunofluorescence (IF), real- time PCR for pneumocystis, bacterial and mycobacterial culture. Nasopharyngeal aspirates were taken for immunofluorescence (IF), real-time PCR for pneumocystis and PCR for respiratory viruses. A blood specimen for bacterial culture and for cytomegalovirus PCR was taken. Children were followed for the duration of their hospitalisation and the outcome was recorded.

**Results:**

202 children [median (interquartile range, IQR) age 3.2 (2.1– 4.6) months] were enrolled; 124 (61.4%) were HIV infected. PCP was identified in 109 (54%) children using PCR, compared to 43 (21%) using IF and Grocott staining (*p* < 0.0001). Most PCP cases (88, 81%) occurred in HIV-infected children. All 21 cases (19%) occurring in HIV- negative children had another risk factor for PCP. On logistic regression, predictive factors for PCP were HIV infection, lack of fever, high respiratory rate and low oxygen saturation whilst cotrimoxazole prophylaxis was protective (OR 0.24; 95% CI 0.1 to 0.5; *p* < 0.002). The case fatality of children with PCP was higher than those without PCP (32.1% versus 17.2%; relative risk 1.87; 95% confidence interval (CI) 1.11 – 3.15). Amongst HIV-infected children, a CD4 less than 15% was the only independent predictor of mortality.

**Conclusions:**

The diagnostic yield for PCP is more than 2.5 times higher on PCR than other detection methods. PCP is a very common cause of severe hypoxic pneumonia and is associated with high mortality in HIV-infected African infants.

## Background

*Pneumocystis* pneumonia (PCP) is a major cause of morbidity and mortality in HIV infected infants [1-5]. Approximately 29 – 67% of respiratory related deaths among African HIV infected children have been associated with PCP [6-8], and in-hospital case-fatality rates range from 20 – 63% [1,2,9-11]. South African studies have reported that the prevalence of PCP ranges from 10% to 49% among antiretroviral naïve HIV infected children hospitalized with pneumonia [9-11]. PCP has also been shown to occur in HIV uninfected infants, mostly with an underlying predisposing factor such as HIV exposure or malnutrition [2,4,12-16].

The use of highly active antiretroviral therapy (HAART) has dramatically reduced the incidence of PCP in developed countries [17-19]. However PCP remains a common cause of hospitalization and mortality in HIV-infected South African children [2,4]. In a recent study, 21% of children admitted to a tertiary paediatric hospital with acute severe hypoxic pneumonia were found to have PCP despite a well- functioning paediatric HIV program [2]. In previous studies, immunofluorescence (IF) or silver staining of respiratory secretions have been used for the diagnosis of PCP. Such methods have been reported to be insensitive for diagnosis, potentially leading to under recognition of PCP [20-24].We recently reported that real-time polymerase chain reaction (PCR) is more sensitive than IF for the diagnosis of PCP when used on lower [induced sputum (IS) or non-bronchoscopic broncho-alveolar lavage (BAL)] or upper respiratory tract [nasopharyngeal aspirate (NPA)] specimens. The yield for PCP from upper and lower respiratory tract specimens was similar by PCR [20].

The aim of this study was to describe the incidence, clinical features and outcome of PCP in children when diagnosed with PCR.

## Methods

This was a prospective study of consecutive children hospitalized for acute hypoxic pneumonia at Red Cross War Memorial Children’s Hospital in Cape Town, South Africa from November 2006 to August 2008. Inclusion criteria were an acute onset of respiratory illness requiring hospitalization, defined as age specific tachypnoea, hypoxia and diffuse lung disease not associated with wheezing [2]. Children were excluded if they had received treatment for PCP in the preceding two weeks, if they had been on PCP therapy for the acute illness for more than 48 hours or if informed consent was not obtainable. The study was approved by the Research and Ethics Committee of the Faculty of Health Sciences at the University of Cape Town. Written informed consent for participation in the study was obtained from each child’s parent or legal guardian.

Clinical, radiological and laboratory data were recorded including symptoms, signs and oxygen saturation. The HIV status of a child (if unknown) was confirmed using whole-blood HIV deoxyribonucleic acid PCR (Amplicor HIV-1 DNA test version 1.5, Roche Diagnostics, GmbH, Mannheim, Germany) in those younger than 18 months, or an HIV enzyme-linked immunosorbent assay (Architect HIV Ag/Ab Combo ELISA, Abbott Laboratories, Abbott Park, IL) in older children. CD4 counts and HIV viral loads were done in all children newly diagnosed with HIV and in those in whom HAART was commenced. A full blood count and serum lactate dehydrogenase (LDH) was done on admission.

An upper respiratory tract (NPA) and a lower respiratory tract (LRT) specimen (IS or BAL in intubated patients) were obtained in a standardized manner, as described previously [25,26]. Respiratory specimens were submitted for detection of *P. jirovecii* by direct IF using a monoclonal antibody (IF: Detect IF PC, Axis-Shield, UK) and Grocott staining. Real time PCR were done on stored histopathological specimens, as described by Samuel et al. [20].

Investigation for additional pathogens included “in-house” respiratory viral shell vial culture and rapid viral antigen detection (murine FITC-conjugated anti- RSV or adenovirus monoclonal, Chemicon, Temecula, CA, USA) on respiratory specimens, bacterial blood culture and bacterial and mycobacterial culture on IS or BAL specimens. Blood specimens were also sent for qualitative whole blood nested cytomegalovirus (CMV) PCR (Super-Therm, JMR Holdings, Kent, UK). CMV pneumonia was defined as positive CMV PCR on a blood specimen as well as detection of CMV on a LRT sample.

Children received standard therapy including oxygen, broad spectrum antibiotics and intravenous cotrimoxazole (trimethoprim-sulfamethoxazole) and oral corticosteroids (prednisone 1–2 mg/kg with tapered doses for up to 21 days) as per national guidelines [27]. Eligible children were started on HAART according to national guidelines [28].

### Statistical analysis

Continuous data were tested for normality using the Shapiro- Wilks test. Descriptive statistics, Mann- Whitney U tests for continuous data, and chi- square tests for categorical data (Yates- corrected chi-square tests where values in the cells were <10) were performed using *STATISTICA* data analysis software system *(version 8, StatSoft, Inc.* 2004). Forward stepwise logistic regression analyses, to determine predictive variables for the dichotomous outcomes of PCP and mortality, were performed using STATA (version 10.0, Statcorp, Texas, USA). Model variables were selected if they were associated with the outcome of interest on univariate analysis and according to biological plausibility. Variables found to be significant when analysed jointly were included in the final model. Weight- for- age and height- for- age Z (or standard deviation) scores were calculated using the Microsoft Office Excel (*Microsoft Corporation 2003*) add-in ImsGrowth Program (*version 2.12, Medical Research Council UK, 2002 – 2005*) (15). The WHO categorises weight- for age and height- for age scores ≤2 as representing moderate under-nutrition and scores ≤3 representing malnutrition [16]. A 95% significance level was chosen.

## Results

Two hundred and two children [92 (45.5%) male] were enrolled with a median (interquartile range, IQR) age of 3.2 (2.1 – 4.6) months. HIV results were available for 200 children; 124 (61.4%) were HIV infected; 34 (16.8%) were HIV exposed but uninfected and 42 (20.8%) were HIV unexposed. Seventy of the HIV exposed or infected children (44.3%) had been in the Prevention of Mother to Child Transmission (PMTCT) program, but only 29 (18.4%) were receiving cotrimoxazole prophylaxis. Five (4%) HIV infected children were on HAART; 72 (58%) were started on HAART during hospitalization, at a median (IQR) of 10 (7 – 14.5) days after admission. One hundred and nine (54%) patients received gancyclovir for presumed or proven CMV infection.

Most children were under- nourished with median (IQR) weight- for age and height- for age Z scores of −2.5 (−4.3 to −1.5) and −2.3 (−3.9 to −0.9) respectively. The most common presenting features were cough (85.1%), vomiting (31.2%), diarrhoea (21.8%), or poor feeding (24.3%); these did not differ by HIV status.

### PCP

*Pneumocystis jirovecii* was detected in 43 (21.3%) children by IF and/or Grocott staining and in 109 (54.0%) children using PCR (*p* < 0.0001) on 107 induced sputum and 97 BAL specimens. No child was positive by IF and negative by PCR. Eighty- seven children (79.8%) with PCP were HIV infected (Table 1). Risk factors amongst the 21 HIV uninfected children with PCP included malnutrition in 12 (57.1%) children, HIV exposure in 5 (23.8%); a history of prematurity in 5 (23.8%); congenital cardiac disease in 2 (9.5%); and primary immune deficiency and post- transplant immunosuppression in one child each. Clinical features distinguishing children with PCP from those without PCP were absence of fever, higher respiratory rate and lower oxygen saturation on admission (Table 1).

**Table 1:** Univariate comparison between PCP positive and negative patients by presenting clinical and laboratory data

**Variable**	**PCP positive**	**PCP negative**	***p***
	**n = 109**	**n =93**	
**Presenting symptoms n (%)**			
Cough	94 (86.2)	78 (83.9)	0.6
Fever	30 (27.5)	47 (50.5)	0.0008
Poor feeding	28 (25.7)	21 (22.6)	0.6
**Patient characteristics**			
Gender M:F	47:62	45:48	0.5
Age (Months)	3.4 (2.7 – 4.0)	2.2 (1.3 – 6.8)	0.03
HIV infected n (%)	87 (79.8)	37 (39.8)	<0.0001
HIV exposed, uninfected	5 (4.6)	29 (31.2)	<0.0001
Weight for age Z score	−2.9 (−4.6 - -1.6)	−2.4 (−3.9 - -1.36)	0.08
Height for age Z score	−2.4 (−4.5 - -0.9)	−2.3 (−3.2 - -0.9)	0.24
Duration of symptoms (days )	3.0 (2.0 – 7.0)	3.0 (1.0 – 7.0)	0.66
**Admission signs**			
Respiratory rate on admission (breaths per minute)	70.0 (60.0 – 80.0)	60.0 (50 – 70)	0.0002
Subcostal recessions n (%)	107 (98.2)	83 (89.2)	0.8
SpO_2_ (%) in room air (n = 146)	77.0 (65.5 – 84.0)	87.5 (75.0 – 92.0)	<0.0001
**Laboratory investigations**			
CD4% in HIV infected children	17.1 (9.9 – 29.7)	21.0 (14.7 – 29.7)	0.12
	n = 80	n = 35	
Lactate dehydrogenase (u/l)	710.5 (534.5 – 1067.0)	350.0 (238.0 – 610.0)	<0.0001
	n = 57	n = 35	

Continuous data are median (interquartile range).

### Co-infection

Co-infection with CMV was common in children with PCP. The prevalence of CMV pneumonia or viraemia was higher in children with PCP compared to those without PCP (Table 2). In contrast other respiratory viruses were more commonly identified in children without PCP (Table 2). There was no difference in the rate of bacteraemia or culture confirmed tuberculosis between the two groups.

**Table 2:** Co-infection in children with and without PCP

**Variable**	**PCP positive**	**PCP negative**	***p***
	**n = 109**	**n =93**	
Cytomegalovirus (CMV) blood PCR positive	81 (74.3)	43 (46.2)	<0.0001
CMV pneumonia	34 (31.2)	11 (11.8)	0.001
*M. tuberculosis* culture positive	1 (0.9)	4 (4.3)	0.3
Viruses other than CMV	29 (26.6)	41 (44)	0.009
Bacteraemia	7 (6.4)	13 (14.0)	0.12
*Staphylococcus aureus*	1	1	
*Escherichia coli*	1	1	
*Klebsiella pneumoniae*	1	1	
*Enterococcus faecalis*	1	1	
*Streptococcus pneumoniae*	0	1	
*Pseudomonas aeruginosa*	0	3	
Non-typhoidal *Salmonella spp.*	0	1	

Numbers are n (%).

### Predictive factors for PCP

LDH and SpO_2_ were not included in the multiple regression analyses due to missing data.In the final model cotrimoxazole prophylaxis was found to be protective for PCP; whilst HIV infection, CMV viraemia, lack of fever and tachypnoea were associated with PCP (Table 3).

**Table 3:** Logistic regression model of predictive factors for PCP

**Variable**	**Adjusted odds ratio**	**95% CI**	***p***
Cotrimoxazole prophylaxis	0.2	0.07 – 0.5	0.002
HIV infection	8.2	3.8 – 17.8	< 0.0001
CMV blood PCR positivity	2.4	1.2 – 5.0	0.02
Fever	0.3	0.4 – 0.6	0.001
Respiratory rate >60 breaths per minute	3.5	1.7 – 7.0	0.0005

CMV-cytomegalovirus; CI- confidence interval. Lactate dehydrogenase and oxygen saturation were not included due to missing data.

### Outcome

There was no difference between children with PCP compared to those without PCP in terms of PICU admission; ventilation requirements and duration; or length of hospital and PICU stay. The in-hospital mortality was 35 (32.1%) in children with PCP compared to 16 (17.2%) in those without PCP (relative risk 1.87; 95% CI 1.11 – 3.15; *p* = 0.02). Multiple regression controlling for age, HIV infection, PCP and cotrimoxazole prophylaxis showed that only HIV infection was predictive of mortality (OR 3.7, 95% CI 1.5 – 9.0; *p* = 0.004). In a separate model of 115 HIV infected children with complete data, only CD4 count <15% was significantly associated with mortality (OR 3.6, 95% CI 1.4 – 9.0; *p* = 0.006) (Table 4). None of the five HIV exposed uninfected infants with PCP died.

**Table 4:** Logistic regression model for predictors of outcome in HIV infected children (n = 115)

	**Adjusted odds ratio**	**95% CI**	***p***
Age (months)	1.00	0.97 – 1.04	0.9
Gender	0.61	0.23 – 1.62	0.3
Weight for age (%)	0.97	0.78 – 1.20	0.8
Cotrimoxazole prophylaxis	1.51	0.47 – 4.83	0.5
CD4 < 15%	3.59	1.43 – 9.01	0.006
PCP	1.12	0.36 – 3.52	0.8
CMV pneumonia	1.92	0.74 – 4.92	0.2

PCP pneumocystis pneumonia; CMV cytomegalovirus.

## Discussion

PCP was diagnosed in more than half of children hospitalised with hypoxic pneumonia using molecular techniques, more than double that diagnosed by IF or Grocott staining on the same respiratory samples [2]. The incidence of PCP reported in prior studies based on IF staining of respiratory secretions may therefore be a large underestimate [1,2,6-10,29-32]. The study indicates that PCP in HIV-infected infants is even more of a concern than was previously recognised, and emphasises the need to strengthen paediatric HIV programs including early use of cotrimoxazole prophylaxis and HAART according to current global guidelines [29,33].

Cotrimoxazole prophylaxis and HAART had been instituted in only a minority of eligible children despite the free availability of HAART and PMTCT programs in this area of South Africa. Reassuringly, cotrimoxazole prophylaxis was found to be highly effective for preventing PCP, supporting the widespread use of this cost-effective intervention [29,34,35]. Although this study was not designed to investigate implementation of national policies for paediatric HIV or adherence to HAART or cotrimoxazole, the results indicate that the HIV program is still not functioning appropriately, with potentially devastating consequences. Further research to investigate the underlying reasons for failure of the PMTCT and HAART programs in this setting is needed.

The in- hospital case fatality rate for children with PCP of 32% is within the range reported in other African studies [1,9-11], and is also similar to that reported eight years previously from the same study site [9].

A minority of HIV uninfected children also developed PCP. Consistent with prior reports, a number of risk factors were identified [1,9,11,36] including malnutrition and HIV exposure [4,12,14,15]. HIV exposed infants may be at increased risk of PCP due to impaired immunity, exposure to *P. jirovecii* from an HIV infected mother or adult, or poor protection from maternal antibodies [4,12,15,37,38]. All HIV exposed but uninfected infants with proven PCP survived, consistent with previous reports of better outcome in HIV uninfected compared to HIV infected infants [16]. With strengthening of PMTCT programs, HIV exposed but uninfected infants are an increasingly important group who may be at risk for PCP. The use of cotrimoxazole prophylaxis in this group deserves further consideration given the increasing number of case reports of PCP in HIV-exposed uninfected infants and this growing population [4,39].

Clinical and laboratory measures associated with PCP were similar to previous reports [2,32]. Although elevated LDH may be a non-specific marker of lung injury [40], these results suggest that there should be a high index of suspicion of PCP in those children with raised LDH admitted to hospital with severe pneumonia, particularly when there are other known risk factors for PCP.

As expected, co-infection with *P. jirovecii* and other pathogens, particularly viruses, was common [2-4,6,41,42]. Similar to previous studies, CMV coinfection occurred most frequently, in a third of children with PCP [4,6,7,43,44]. This may also reflect severe immunosuppression as CMV pneumonia has been associated with moderate or severe immunosuppression in HIV-infected infants [4-6,43-46].

Limitations of this study include lack of a control group. Several adult studies have reported carriage of *P. jirovecii* as detected by a positive PCR on respiratory specimens [22,47,48], however, positive PCR results in our study are unlikely to represent colonisation given the presentation of severe pneumonia, the young age of infants and the protective effect of cotrimoxazole prophylaxis [23]. We were unable to use response to treatment as a means of confirming PCR diagnosis as all children were treated for presumptive PCP based on clinical signs. The lack of a gold standard for PCP diagnosis makes testing of any new diagnostic modality challenging, especially as the organism cannot be easily cultured. This was a single- centre study of a selected population admitted to a tertiary hospital; therefore results may not be generalisable to children from different geographical areas and with different clinical disease spectra. Further studies of PCR based diagnosis in children with non-HIV immunosuppression are needed.

## Conclusions

PCP remains a common cause of severe hypoxic pneumonia and is associated with high mortality in HIV-infected African infants. New molecular diagnost ic methods indicate that the burden of PCP in this populat ion has been under -est imated in the past.

## Competing interests

The authors declare that they have no competing interests.

## Authors’ contributions

BM recruited patients, acquired and analysed data, and drafted the manuscript. HZ was responsible for study conception and design, clinical supervision and obtaining funding. AW was the laboratory supervisor, contributed to the study design and obtained funding. MZ recruited patients and acquired data. CS performed the molecular investigations. All authors contributed to the final manuscript and have read and approved of it.
